# Platelet lysate for the treatment of osteoarthritis: a systematic review of preclinical and clinical studies

**DOI:** 10.1007/s12306-024-00827-z

**Published:** 2024-06-03

**Authors:** K. Valtetsiotis, A. Di Martino, M. Brunello, C. D’Agostino, R. Poluzzi, R. Ferri, P. Mora, F. Traina, C. Faldini

**Affiliations:** 1 Department of Biomedical and Neuromotor Science-DIBINEM, University of Bologna, 40127 Bologna, Italy; 2 1st Orthopedic and Traumatology Department, IRCCS Istituto Ortopedico Rizzoli, Via Giulio Cesare Pupilli 1, 40136 Bologna, Italy; 3 Orthopedics-Traumatology and Prosthetic Surgery and Hip and Knee Revision, IRCCS Istituto Ortopedico Rizzoli, 40136 Bologna, Italy

**Keywords:** Knee, Platelet lysate, Osteoarthritis, Injection

## Abstract

Intra-articular injection-based therapy is often used aside conservative treatment and lifestyle modifications to manage knee osteoarthritis (KO) patients. Conventional injections contain steroids and hyaluronic acid, while more recently multipotential adult stem cell, platelet-rich plasma (PRP), and platelet lysate (PL) injections have been used to promote cartilage regeneration or repair. The aim of the current study is to analyse current evidence on PL injections for the treatment of KO and to determine if these are effective and how these perform compared to other injection regimens. The databases of Scopus, Embase, PubMed, Web of Science, and Cochrane Library were searched on 30 June 2023. Risk of bias was assessed using the SYRCLE tool for animal studies and Cochrane RoB 2 as well as ROBINS-I tool for human studies. Studies were included if these were in English, any year, and regarded animals with osteoarthritis (OA) or human adult patients with OA. In vitro trials and non-adult human studies were excluded. Results on OA symptom stage and severity, and pain were recorded. The research retrieved three human studies (*n* = 48, *n* = 25, *n* = 58) and four animal studies: one rabbit, two studies, and one rat study. PL was found to decrease KO symptoms at follow-up ≤ 1 year with respect to baseline levels and when compared to hyaluronic acid or platelet-rich plasma. Symptoms returned 6 months–1 year after the final administration, with studies showing peak efficacy at approximately 6 months. Animal studies showed clinical improvements, reduction of lameness, and partial effect on the cartilage regeneration of the seven studies, two had a high risk of bias, four were associated to some concerns, and one had low risk. A major source of bias in these studies was the use of questionnaires and scoring that could be subject to interpretation. Overall, PL was well-tolerated and showed efficacy comparable to PRP; when pain control was assessed, it showed similar efficacy compared to hyaluronic acid. These findings may support its use in clinical trials to confirm these initial findings; future research should also focus on the comparison with other non-surgical treatments, on a more detail of the potential regenerative properties, and to optimise the treatment schedule.

## Introduction

Osteoarthritis (OA), also known as degenerative arthritis, is the most common form of joint disease, and it affects more than 1 in 4 adults in the US [1, 2]. Although disease progression is associated with an increase in pain stemming from the joint, it does not correlate to the severity of OA due to structural differences among patients in the entity of joint degradation and for the nociceptors that are specifically activated by the mechanical stimulus [3]. Knee OA is the most common type (6% prevalence in adults) [4], and in 2020, it was estimated that approximately 654.1 million individuals suffered from knee OA worldwide. This accounts for a 16% global prevalence rate in individuals over 15 years of age and a 22.90% prevalence in individuals over 40 years of age [5]. Between 2005 and 2015, a 34.6% increase in the years lived with disability was observed in patients with knee OA [6, 7]. Its clinical presentation is heterogeneous, with typical symptoms including pain, stiffness, and movement restriction [8].

Treatments of knee osteoarthritis (KOA) vary according to the severity of the disease: the milder the symptoms, the milder the medical approach. In the initial stages, it may be managed by lifestyle changes, such as weight loss, and conservative treatments including therapeutic physical exercise, aerobic exercise, balneotherapy, hydro-kinesitherapy, muscle strengthening exercise, mind–body exercises, and balance training [9, 10]. Non-pharmacological treatments, such as physiotherapy, are upgraded to a more pharmacological-focused management of symptoms in moderate–severe cases, ending up with surgical intervention for the advanced stages [11].

To prevent or slow KOA progression, some regenerative solutions have been developed to be used in the treatment of moderate cases [12]. In the past few years, platelet-rich plasma (PRP) gained popularity due to its chondroprotective properties, useful in the treatment of symptomatic KOA [13]. Recently, platelet lysate (PL), a new platelet derivate, was used in stem cell research as a culture medium for mesenchymal stem cells in the treatment of OA and other diseases [14, 15]. Autologous PL derives from plasma extracted from a patient, and subsequently, the platelets undergo lysis, so that a cell-free extract is created. Its injection has been proposed for theoretical regenerative properties due to its high number of growth factors, chemokines, cytokines, and proteins that promote on-site healing [16]. Compared to PRP, it is less temperature-sensitive, allowing for cryopreservation and long-term storage [16]. It may overcome some PRP disadvantages, including the presence of contained growth factors not being released, and a high variability in platelet and growth factor quantity due to the multiple preparation methods and differences among donors [17]. PL may, therefore, find routinary use in the management of KOA; consequently, it is important to determine its safety, efficacy, and if it works distinctly better compared to PRP.

This study aims to determine the efficacy of PL injections for the treatment of KOA and to assess how it compares to other injection treatments. The literature review aimed to clearly summarise the existing knowledge from basic research studies, evaluate the strengths and weakness of the retrieved articles, and provide ideas for the development of further studies. To the best of our knowledge, this is the first systematic review examining the effect of PL on KOA. This review will be answering the questions: (1) *Are PL injections an effective treatment for KOA?* and (2) How do *PL injections compare with PRP for KOA?*

## Methodology

Inclusion criteria were peer-reviewed studies in English, any year, full-text available, for animal models, animals with OA, and for human studies, adult patients with OA. Exclusion criteria were: studies in other languages, abstract only available, in vitro trials, and non-adult human studies. Studies were grouped in two blocks: animal studies (*n* = 4) and human studies (*n* = 3).

The search query used for all databases was: (osteoarthritis* OR osteoarthrosis OR “degenerative arthritis*”) AND platelet AND (lysis OR lysate). Results were filtered to English only articles.

**Fig. 1:**
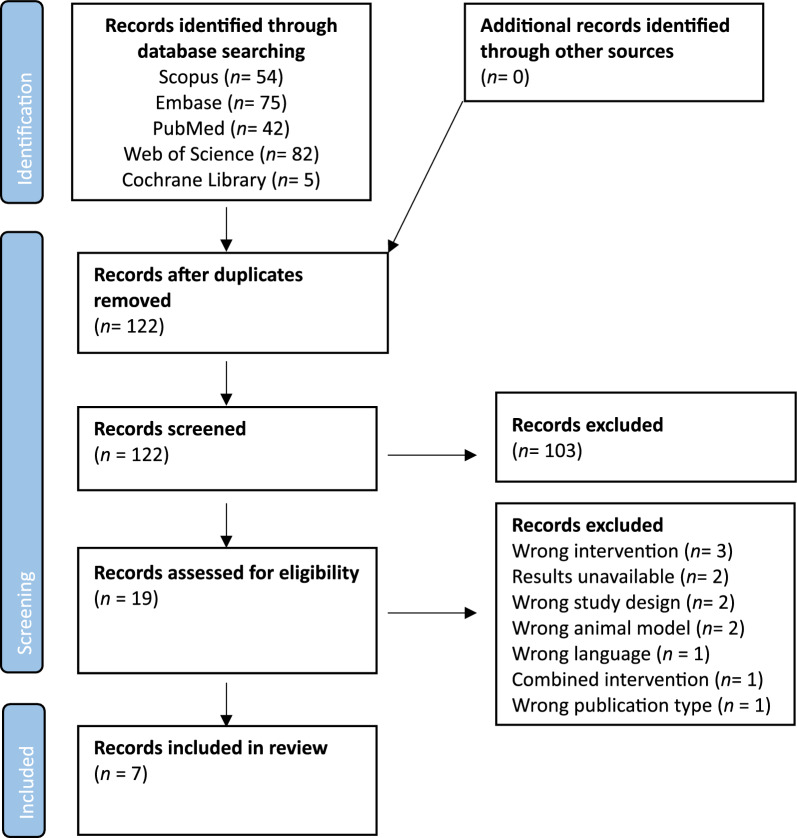
Flowchart of screened and accepted studies. Databases were last searched on 30 June 2023

To assess if the results met the inclusion criteria, results were first filtered to eliminate duplicates. Duplicate identification was performed automatically using the free web tool *Rayyan.ai*, though any potential duplicates were then manually confirmed by a researcher (KV). Afterwards, two independent researchers (KV and MB) screen by their title and abstract only, quickly removing any records out of the inclusion criteria. They then continued by assessing the eligibility of the remaining records by a full reading of the paper. In the end, results were compared to identify any discrepancies between the two researchers. In case of doubt, a senior researcher (ADM) expressed over the inclusion of the manuscript. In Fig. 1, a flowchart is available demonstrating the agreed upon screened and accepted articles (Table 1).

**Table 1:** SYRCLE’s risk of bias tool for animal studies

Study	Study type	Selection			Performance		Detection		Attrition	Reporting	Other	Overall bias
		Sequence generation	Baseline characteristics	Allocation concealment	Random housing	Blinding researchers	Random outcome assessment	Blinding assessors	Incomplete outcome data	Selective outcome reporting	Additonal risk present	
Hsieh et al. [19]	Rabbit study	+	+	?	+	−	?	−	+	+	−	Some concerns
Perrone et al. [20]	Equine study	?	+	−	?	−	?	−	+	+	+	High
Tyrnenopoulou et al. [21]	Equine study	+	+	−	−	−	?	−	+	+	?	High
Yan et al. [22]	Rat study	+	?	+	−	+	−	+	+	?	+	Some concerns

The study by Li et al. [18] appeared at first to meet all the inclusion criteria, but it was excluded during the study selection process. Although the study tested PL efficacy, the formulation creation involved the usage of biological microspheres loaded with PL. A large part of the study involved the creation of creating a good microsphere vehicle for the PL, with PL not being tested outside of the microspheres. This delivery method played a large role in the efficacy of the results and as such we did not consider it appropriate to include it given that this additional factor influences results too much. As such, it would not be possible to draw conclusions on the usage of PL without microspheres from this study (Table 2).

**Table 2:** Cochrane risk of bias 2 tool for randomised controlled studies [26]

Author	Randomisation process	Deviations from intended interventions	Missing outcome data	Measurement of outcome	Selection of reported result	Overall bias
Hosseini et al. [24]	Low	Low	Low	Low	Some concerns	Some concerns
Lisi et al. [25]	Low	Low	Low	Low	Low	Low

Data collection was performed manually, with one researcher (KV) collecting pertinent quantitative or qualitative data. All studies were conducted for periods of a year or less (Table 3).

**Table 3:** ROBINS-I tool for non-randomised studies [27]

Author	Confounding	Selection bias	Bias in measurement classification of interventions	Bias due to deviations from intended interventions	Bias due to missing data	Bias in measurement of outcomes	Bias in selection of the reported result	Overall bias
Al-Ajlouni et al. [23]	Low	Low	Low	Low	Low	High	Low	Some concerns

Research outcomes included any assessment that examined (a) stage and severity of OA symptoms and (b) pain. Data were sought for all characteristics and all in vivo studies. Studies with missing data were assessed on a case-by-case basis, and missing data were commented on and considered in forming the risk of bias. The risk of bias was assessed using three tools; 1. Cochrane RoB2 for RCTs, 2. ROBINS-I for the non-randomised human trial, and 3. SYRCLE for the animal studies. The risk of bias assessment was performed independently by two assessors (CD and RP), and the results were compared, with any discrepancies discussed, and the final assessment agreed upon mutually. For the outcome of pain, KOOS score, VAS, WOMAC, range of motion, Lysholm score, Tegner score, AKSS score, Lequesne score, AEEP lameness score, mechanical withdrawal threshold (MWT), thermal withdrawal latency (TWL), spontaneous activity, treadmill gait analysis, and total paw area were used to measure the state of the joint and pain. Results of the studies were provided in a quick to read summary table (Table 4) and then further elaborated in the results section. The confidence given to the body of evidence of an outcome was based on the risk of bias.

**Table 4:** Summary of the results

Authors	Date published	Type of study	Type of OA	No. of patients/subjects	Length of follow-up	Method & analysed variables	Results
Hsieh et al. [19]	12/2/2023	Rabbit study	Simulated OA by surgically creating an osteochondral defect in hind knees	3	12 weeks	Rabbits administered PL or saline in knees with osteochondral defect. ICRS scoring of repaired cartilage based on macroscopic evaluation, histological assessment	ICRS score: 11.7 control; 19.4 experimental group. Histological evaluation of osteochondral defects score: 7.8 control; 9 experimental group
Tyrnenopoulou et al. [21]	1/2/2016	Equine study	Naturally occurring OA of the distal extrapharyngeal joint	15	1 year	OA horses received either PL injections or saline injections. Analysis: AAEP grade of lameness	After 2nd injection (3 weeks) PL group had significantly lower lameness scores versus control (*p* < 0.0005)
Perrone et al. [20]	1/2/2020	Equine study	Horses with OA in intertarsal joints	44	60 days	OA horses were administered PL injections. Analysis: AAEP grade of lameness	AEEP score significantly lower (*p* < 0.01) compared to control at days 30 and 60
Yan et al. [22]	14/5/2019	Rat study	Induced OA through MIA injection	50	4 weeks	Rats administered placebo, or low/medium/high doses of PL. Analysis: mechanical withdrawal threshold (MWT), thermal withdrawal latency (TWL), spontaneous activity, treadmill gait analysis, total paw area, and semiquantitative positive area analysis for Col2 and Mmp13	Significantly improved (*p* < 0.01) MWT, TWL, spontaneous activity, treadmill gait analysis, and total paw area in PL-H group versus control. Significantly (*p* < 0.01) higher Col2 area and significantly (*p* < 0.01) lower Mmp12 area in PL-H versus control, indicating reduced OA effects
Al-Ajlouni et al. [23]	1/11/2015	Open-label prospective study	Adult knee OA	48	52 weeks	OA patients administered PL in knee joint. Analysis: KOOS scoring	Symptom score decreased from 11.1 at baseline to 8.7 at 52 weeks (*p* < 0.0001). Stiffness score decreased from 2.2 at baseline to 1.6 at 52 weeks (*p* < 0.016). Pain score decreased from 14.2 to 9.2 by week 52 (*p* < 0.0001). Daily living score improved from 25 to 18.7 (*p* < 0.0001). Sport score improved from 10.7 to 8.1 (*p* < 0.0001*)*
Hosseini et al. [24]	23/6/2023	Randomised clinical study (RCT)	Adult knee OA (female)	25	6 months	OA patients administered PRP in one knee joint and PL in the other. Analysis: VAS questionnaire, WOMAC questionnaire, and range of motion (ROM)	At 6 months, PL performed significantly better versus PRP at: VAS grade (1.088 ± 0.265 vs. 2.136 ± 0.464, *p* = 0.0384), ROM (133.76 ± 5.077 vs. 131.32 ± 3.532; *p* = 0.0314), WOMAC function (14 ± 3.808 vs. 20.44 ± 5.331; *p* = 0.0438), WOMAC pain (2.28 ± 1.514 vs. 4.52 ± 1.96; *p* = 0.0237), and WOMAC stiffness (1.08 ± 0.812 vs. 2.08 ± 1.288; *p* = 0.0206)
Lisi et al. [25]	7/7/2017	Phase 2 RCT	Grade II/III OA in adults	58	1 year	Patients were treated with either hyaluronic acid or PL. Analysis: VAS, WOMAC, Lysholm, Tegner, AKSS, and Lequesne scoring	MRI results were significantly better at 6 months in PL group versus HA group (51.6% PL vs. 25.8% HA; p = 0.038). At 12 months, PL group score significantly better at WOMAC ADL and Lequesne scoring

### PL preparation

The various ways of LP preparation of the following studies were collected, meticulously describing the various steps. Hsieh et al. [19] prepared the PL with the porcine whole blood that was centrifuged, and the supernatant was taken, leucocytes were filtered, platelets were lysed by the freeze–thaw method, heated to remove the complement, and filtered with a 0.22-μm pore size filter.

Perrone et al. [20] prepared the PL with 8 mL of blood collected in a tube containing 2 mL of trisodium citrate as anticoagulant. Blood samples were first centrifuged for 10 min at 1000*g*. The supernatant was further centrifuged for 10 min at 1500*g*. One mL from the lower portion of the suspension was then transferred to an empty tube. Three cycles of freezing and thawing were performed, and the resulting suspension was sterilised by filtration with a 0.22-mm filter. Before starting the freeze–thaw cycles, a platelet count was carried out in a Neubauer chamber using a commercial kit. The platelet count ranged from 400,000 to 700,000 per mm^3^.

Tyrnenopoulou et al. [21] made the PL with 50 millilitre of whole blood, collected aseptically from the left jugular vein of each horse of the PL group, via an 18G needle, and this was placed in vials containing about 6 ml of an anticoagulant solution (citrate phosphate dextrose). The samples were centrifuged, initially at 270×*g* for 7 min and then at 1000×*g* for 5 min, the supernatant plasma just above the buffy coat was separated carefully to avoid leucocyte aspiration, and a mechanical activation of platelets by a freeze-thaw process (frozen in − 80 °C for 30 min and then thawed at room temperature) was performed. The final volume of PL obtained from each horse ranged from 3 to 7 ml.

PL was obtained by Yan et al. [22] lysing platelet concentrates of rat. Briefly, anticoagulant whole blood (3% *v*/*v* sodium citrate) was treated by sequential rounds of centrifugation at 4 °C (10 min at 210×*g* and 5 min for 210×*g*), with the non-erythrocyte volume collected subsequently to each round. The collected buffy coat was washed three times with phosphate-buffered saline and concentrated through supernatant removal to obtain platelet concentrates. The final platelet number was measured and standardised to 1 × 10^8^ platelets/ml. The platelet concentrate was lysed by repeating a freeze–thaw (− 80 °C–37 °C) three times, followed by centrifugation at 2000×*g* for 10 min to remove remaining platelet fragments. The obtained supernatant containing bioactive growth factors (PL) was divided into aliquots and stored at − 80 °C before use.

Al-Ajlouni et al. [23] prepared the PL from blood samples that were obtained in sterile citrate tubes on the day of intra-articular injection. A total of 20 mL of blood were collected in each occasion. The autologous blood was centrifuged at 1000 rpm for 13 min to obtain PRP. A second centrifugation was done for the PRP at 4000 rpm for 10 min to obtain platelet pellet and supernatant platelet poor plasma (PPP). The pellet was resuspended in 5 mL of PPP. The suspended pellet was frozen twice at − 80 °C, each time for 10 min. The suspension was thawed between the first and second freeze and after the second freeze. The thawed suspension was centrifuged at 4000 rpm, and the supernatant was obtained and filtered with 0.2-μm filters. The filtered product was drawn in a sterile syringe and used for intra-articular injection. Platelet count in the initial blood sample and the PRP sample were obtained.

Hosseini et al. [24] prepared PL from 20 mL of blood, which was drawn from each patient in sterile citrate tubes. After serial centrifugation, the platelet pellet was resuspended in physiologic saline (0.9% sodium chloride) in order to eliminate likely contaminants with plasma reaching a final platelet concentration of about 1 × 10^7^ platelets/μL. To activate platelets, the suspended pellet (PRP) was subjected to two sequential freeze/thaw cycles (freezing at − 80 °C, each time for 60 min, rapidly thawing at 37 °C for 15 min) to lyse the platelets to obtain the released growth factors. To remove platelet bodies or debris, the thawed suspension was centrifuged at 400*g* for 20 min at room temperature, and the supernatant (PL), containing the cocktail of factors released by the platelets, was harvested and filtered with 0.45- and 0.22-μm membrane filter. Heparin (2 U/ml) was added as an anticoagulant to filtered PL and divided into aliquots and stored at − 80 °C until use.

Lisi et al. [25] prepared the PL with 20 mL of autologous whole blood that was sampled from each patient and 2 mL of anticoagulant citrate dextrose solution, added directly through the syringe as anticoagulant; finally, the vial was gently centrifuged at 900 r/min for 7 min. Platelet-rich plasma was collected.

## Results

### Selection results

Manuscripts were published between 2015 and 2023. According to the Cochrane Risk of Bias 2 Tool for Randomised Controlled Studies, one reported as an overall judgement “Some concerns” [24] an another “Low” [25]; with the ROBINS-I tool for non-randomised study obtained a judgement of “Some Concerns” [23]. The SYRCLE evaluating score system for animal studies showed that one study achieved 3/18 points [20], other one achieved 4/18 points [21] another 5/18 points [19] and 6/18 points [22]. The animal studies presented had methodological limitations. These were observational studies with no rigid selection criteria, and the SYRCLE score demonstrated significant shortcomings in both performance and detection (Table 1). Human studies presented a methodology with several criticisms as highlighted in their scores (Tables 2 and 3). Therefore, to correctly interpret data, these parameters should be considered.

### Included studies

#### Animal studies

Hsieh et al. [19] tested the safety and efficacy of exogenous PL in vivo a randomised controlled trial. PL was derived from porcine blood that was centrifuged, filtered, and lysed. PL was to be administered to OA rabbits on the knee. First, an experiment was run to determine if the PL solution does not in itself cause inflammation and thus to determine its safety. A total of three rabbits were included in the study, they had one hind knee joint injected with PL and one with saline, as a control. All rabbits but one showed no sign of inflammation. The researchers noted that the one rabbit with an inflammatory reaction in the PL-injected knee may have had an allergic reaction to the PL or sustained injuries while it was being injected. The inflammatory reaction was noted in the cross-section and was not otherwise visible. Once the safety experiment was finished, three other rabbits were chosen to test the efficacy of PL in healing osteochondral damage. A 4 mm^2^ osteochondral defect in a randomly allocated hind knee joint was created, and the three rabbits were injected with PL (0.5 mL with a 25 ng nct) in both knees at: 1. The day post-operation, 2. the 2nd week, and 3. the 4th week. After 12 weeks had elapsed, the rabbits were sacrificed. The experimental group had a higher ICRS score (19.4 vs. 11.7) and a better histological evaluation (9 vs. 7.8) when compared to the control. None of the joints in the experimental group displayed signs of traumatic arthritis. On the other hand, all three joints in the control group did display such signs. Overall, cartilage regeneration was somewhat better in the experimental group—though the lack of statistical evidence, combined with a medium risk of bias awarded this study low confidence. This was the first in vivo test of exogeneous PL.

Tyrnenopoulou et al. [21] examined the effect of PL on equines. Fifteen adult equines were randomly allocated to either a control group (*n* = 5), receiving 3-mL saline injections, or a PL group (*n* = 10), receiving 3-mL autologous PL injections in the distal interphalangeal joint. The horses received two injections, one at the beginning of the experiment and one after 3 weeks. Ten days after the second injection, there was a significant difference in the crease of lameness scores between the PL groups and the control (*p* < 0.0005). In fact, 1 horse showed a slight increase, 2 notable improvements, and 7 had a complete elimination of lameness. In the 6-month follow-up, the radiographs revealed were no change in the OA since the start of the experiment. However, past the 6-month lameness started to gradually return, and by the 1 year follow-up, all horses were back to their pre-experimental scores. Due to the high risk of bias assigned as explained in the discussion, a low confidence was given to this study as well.

Perrone et al. [20] evaluated 23 horses, 15 males and 8 females in vivo a randomised controlled trial, all of which showed that OA in the intratarsal joints was administered PL for a period of 60 days. Synovial fluid samples were taken at baseline (day 0), days 10, 30, and 60. As a control, a group of 21 healthy horses was selected and had synovial fluid samples taken at the same interval. The autologous PL was formulated and found to have a platelet count between 400,000 and 700,000 mm^3^. The PL was filtered to ensure a low quantity of leucocytes, a potential source of adverse events. The clinical score based on the AAEP lameness score was significantly lower (*p* < 0.01) at day 30 and day 60 when compared to control. Between the two, there was no significant difference, suggesting that the peak effects are reached around day 30 and the score then stabilises. MMP-9, which was only found in OA horses, decreased significantly on day 10 compared to baseline (35.68 ± 11.03% vs. 100 ± 28.33; *p* < 0.05). However, no significant difference was found compared to baseline on days 30 and 60. ADAMTS-5 is a protease, its levels have been found to increase in OA cases [28]. ADAMTS-5 decreased significantly dates 10, 30, and 60 when compared to control (*p* < 0.05 for days 10 and 30, *p* < 0.01 for day 60). Glycosaminoglycan (GAG) levels have also found to be decreased in horses with OA. Their values increased significantly in the PL group at days 10 and 30 when compared to control. However, by day 60, there was no longer a significant difference, suggesting that its duration of increase is short. Due to the high risk of bias, the results of this study were given a low confidence.

Yan et al. [22] conducted an experiment to test the efficacy of PL in simulated OA in rats. A total of 50 rats were selected for the study, they were randomly placed into one of the five groups: normal control group (NC, administered saline), model OA group (model), low-dose PL (PL-L, 10^5^ platelet-derived PL), medium-dose PL (PL-M, 10^6^ platelet-derived PL), and high-dose PL (PL-H, 10^7^ platelet-derived PL). All groups besides NC received a 50-μl monoiodoacetate (MIA) dose intra-articularly, triggering cartilage degeneration and OA. After a week, the PL groups received their respective doses through an intra-articular injection, while the NC groups received a saline injection. This was repeated weekly for a total of 4 weeks, after which the rats were analysed and sacrificed for further analysis. By the end of the experiment, all PL groups had significantly lower Mankin’s and OARSI scores compared to the OA model, with progressively better scores in higher concentrations—the PL-H always performing best (Mankin’s: ~ 5 PL-H vs. ~ 7 model; *p* < 0.01. OARSI: ~ 0.25 PL-H vs. ~ 4.25 model; *p* < 0.01). The PL-H group had a better MWT score against the OA model (~ 480 g PL-H vs. ~ 350 g model; *p* < 0.01. A pain resistance indicator) as well as TWL score (~ 9 s PL-H vs. ~ 7 s model; *p* < 0.01. A pain resistance indicator), spontaneous activity (~ 105*n* PL-H vs. 55*n* model; *p* < 0.01. An indicator of wellness), total paw area (~ 500 cm^2^ PL-H vs. ~ 300 cm^2^ model; *p* < 0.01. An indicator of good gait pattern), and unit stride length (~ 0.7 PL-H vs. ~ 0.55 model;* p* < 0.01. An indicator of good gait pattern). Thus, all pre-euthanasia experiments demonstrated a significant improvement in the mice treated with PL versus the OA model. The above results were further supported by the immunohistopathological observation and the semi-quantified positive area of Col2 and Mmp13 (measured in pixel*10^5^), which was done through an immunoreactivity test on the tissue used for the immunohistological analysis. Col2, a gene that is typically supressed in OA [29], had a significant upregulation in the PL groups when compared to the OA model (~ 6.75 PL-H vs. ~ 4.5 model; *p* < 0.01). Furthermore, Mmp13, a gene that is typically promoted in OA, had a significant downregulation (~ 1 PL-H vs. ~ 15 model; *p* < 0.01 [30]). Histopathological staining of articular samples from the knee joints showed that compared to the OA control, increasing the PL dose led a higher number of chondrocytes, a larger mass of matrix collagen, and an increased cartilage surface. An in vitro immunohistological observation revealed a significant (*p* < 0.01) upregulation of genes typically supressed in OA (Col2, aggrecan) and a significant (*p* < 0.01) downregulation of genes typically promoted in OA (Col10, Mmp13, Adamts5, and Adamts9). This provides some context on the potential mechanisms that PL exerts its effects. Overall, this study was found to have a medium level on confidence based on its risk of bias and quantitative data.

#### Human studies

Al-Ajlouni et al. [23] were the first to test autologous PL in humans. The trial included 48 patients of 35–70 years of age that had chronic pain and swelling of one or both knees. PL was administered on days 0, 21, and 42 of the experiment (3-week intervals). The patients had follow-up at 6 months and at 1 year after the procedure. As shown in Table 3, the self-assessed scores of all KOSS measurements (symptoms, stiffness, pain, daily living, and sport) decreased significantly by the 1-year mark. The average of the sum of all scores decreased from 74 ± 19.7 to 52.6 ± 16.97, a significant (*p* < 0.05) decrease. In fact, all patients showed significant (*p* < 0.05) decrease in all scores both at the first assessment (week 32) and the final assessment (week 52), with all the scores further decreasing significantly between the two assessments (*p* < 0.01). This study was given a low confidence score due to its risk of bias, and the way the study was conducted, which is elaborated in the discussion.

Hosseini et al. [24] in a recent article performed an RCT comparing the efficacy of PRP and PL. The study consisted of 25 female patients between the ages of 38–67, suffering from OA in both knees. They were administered PRP injections on one knee and PL injections on the other. The autologous PRP prepared had an average increase of 4.2–4.6 times the platelet counts at baseline. To eliminate any adverse reactions due to the high leucocyte concentration [31], a leucocyte poor PRP was used. The injections were administered three times, with a 21-day interval, and the patients had a 6-month follow-up. The patients were assessed for their range of motion and completed the VAS and WOMAC questionnaire under the care of a blinded-to-intervention physician. At 6 months, the results from the PL knee had significantly better values (*p* < 0.05) at the VAS pain score as well as a higher range of motion. Additionally, the WOMAC scores were significantly higher in all sections: stiffness, pain, and function, as well as in the total score (*p* = 0.033). This study was given medium confidence in its results.

The study from Lisi et al. [25] is the only human RCT currently available. They conducted a Phase 2 RCT testing the efficacy of PL versus hyaluronic acid (HA) in a double-blind trial. Thirty patients were injected thrice with either PL or hyaluronic acid, at 4-week intervals. Hyaluronic acid was at a concentration of 20 mg/2 mL with 5 mL being injected. No side effects were observed in either group. At 6 months, MRI results (the primary outcome) showed a significant difference in PL versus hyaluronic acid group, with PL scoring much higher (51.6% PL vs. 25.8% HA; *p* = 0.038). At 12 months testing of the secondary outcomes, there was no significant difference between PL and HA in VAS score, WOMAC pain, WOMAC rigidity, WOMAC total, AKSS, Lysholm, Tegner, or flexion. However, PL outperformed HA in WOMAC ADL (PL 10 vs. HA 3; *p* = 0.002) and Lequesne (PL 3.5 vs. HA 1.5; *p* = 0.04). This was a well-performed study, and its results were given a high confidence.

## Discussion

The main findings of this systematic review include encouraging results in animal studies, where it is shown that the use of PL on horses, rabbits, and rats led to clinical improvements, reduction of lameness, and partial effect on the cartilage regeneration. Human studies showed a potential role in the conservative management of knee OA with diminished symptoms after interval injections, and improvement in pain scores, range of motion, and WOMAC scores. Currently, however, there are a small number of papers in the literature that do not currently support the use of PL in clinical practice, thus necessitating numerous high-level studies to confirm its therapeutic potential.

Regarding animal studies, Hsieh et al. [19] found that in treated subjects, cartilage regeneration was somewhat better, and the researchers noted that it was primarily fibrocartilage repair and “incompletely differentiated hyaline cartilage”. The treatment was determined to be effective in delaying arthritis (no experimental group joint displayed arthritis) but not at cartilage repair. The exact concentration of PL was not determined. TFG-β, which is one of the six growth factors found in the porcine PL, was used as a determinant of concentration. This was done by measuring its concentration in the created PL and then diluting the PLs concentration so as to achieve a TFG-β total amount of 25 ng, using a standard curve. Compared to the concentration on the PRP, this value tends to vary and that is one of the primary reasons for investigating PL [32], were it may be better controlled. However, it would have been interesting to note the resulting concentration of the other five factors in the final solution so as to draw further conclusions once more in vivo studies are out. This study was likely the first that tested the safety and efficacy of xenogeneic PL in vivo. While the results were modest, these provided the earliest evidence that xenogeneic PL is a safe and effective treatment for cartilage repair, even when the source of origin is another mammal. Finally, there was no statistical analysis done, so the confidence on the value results is constrained. When it comes to the safety of PL, this study had 3 subjects (6.25%) experience intra-articular bleeding. Although none experienced a critical case, the figure is high enough to consider the risks of the treatment. It may have been due to the injection method/technique and not due to PL in itself.

The results of the Tyrnenopoulou et al. [21] study were in line with the results obtained from Hosseini et al. [23] which commented that at 6 months is the peak effect of PL efficacy, the results gradually fading afterwards. This, on the other hand, contradicts the results obtained by Al-Ajlouni [23], who noted that there was a further significant decrease between the 6th and the 12th month of follow-up. More research is still required on the length of effect of PL, so the optimal window for a booster dose may be determined. This study had two major weaknesses. The researchers were not blinded, and the quantitative results relied on an assessment that could be prone to bias in reporting the results. It also did not include any “harder” statistical evidence, e.g. range of motion, which would have been useful to substantiate the assessment results.

The results of the Perrone et al. study [20] complemented the results of Tyrnenopoulou et al. [21]. Perrone et al. found that the maximum decrease in lameness (and thus OA symptoms) occurred at 30 days. This is in line with Tyrnenopoulou, who found a significant difference versus the control at 3 weeks. Therefore, it appeared that the results of PL administration were fast-acting, showing efficacy at around a month post-treatment initiation. An important limitation of this study is that the horses had some heterogeneity in symptoms and severity due to their OA being natural, which may have influenced results.

Yan et al. [22] had the animal study with the least risk of bias, and its results were judged to have medium confidence. Although well conducted, the quantitative results were not given separately and good only be visualised in graphs. As such, the results included in this review are estimates. Even though the significant differences were provided, a supplementary table with raw data would have been best. The PL concentration was found to have a slight effect on the scoring of the groups, with higher concentrations having better results. However, the difference was not found to be statistically significant. This may suggest that an even higher concentration could have yielded better results. Further research should analyse if this may be the case and if there is an increased adverse effect risk with higher concentrations (assuming a low in leucocyte lysate, which as discussed tends to have fewer complications).

As regards human studies, Al-Ajlouni et al. [23] showed some promising early results for the usage of autologous PL in humans. The average platelet concentration was recorded, noted to be 5.6 times higher than the regular whole blood platelet count, though the figure was an average. The researchers could have used a single growth factor from the whole blood sample as an indirect marker of the concentration and subsequently ensure that the final autologous PL for each patient had the same value. The researchers did not provide the values of each individual concentration and the variance between them; thus, it is unclear by how much the platelet concentration varied between prepared PLs. For thrombocytes, a range of thrombocyte counts in the PL is given, with a minimum of 1000 and 1700 × 10^9^/L. This is a very wide range and ideally the counts would have been standardised in some way. Three of the 48 patients had adverse reactions, experiencing intra-articular bleeding, of which two required overnight hospitalisation for monitoring. All made a full recovery. A major limitation of the study was that a control group was not included. Including one—and performing a double-blind trial—would have significantly benefited the authority of the results, as the KOSS is a self-administered questionnaire. The current results have a high risk of being influenced by the placebo effect. This study would have benefited perhaps from being self-controlled by selecting exclusively patients with double knee OA and treating one knee with PL and another without. This study although low in result confidence shows the potential of autologous PL as a safe and efficacious regenerative OA procedure.

Hosseini et al. demonstrated that PL may in fact perform better than PRP in patients with knee OA [24]. Interestingly, the significant difference in scores for PL versus PRP were noted only at 6 months—none of the tested values in fact had a significant difference between PL and PRP at the 1-month assessment. This should be considered when designing future studies to test the efficacy of PL, as its results appear to require a long-time span to show its potential. The timeframe agrees with study of Tyrnenopoulou et al.; the highest efficacy is presented at 6 months; hence, future medical applications of PL should likely be performed bi-annually to maintain a consistent high efficacy. Of course, this would require longer studies that demonstrate the efficacy of such injections beyond 1 year. This study had limited bias and results directly showing the potential for PL to have higher efficacy than PRP.

Lisi et al. were the only RCT available of PL [25], and its results compared to other trials. The study was well conducted with a low risk of bias. One limitation to be noted however, was that although the participants and researchers were blinded, the administrators were not themselves blinded, even though the injecting procedure was the same for both groups. It would have been interesting if MRI scans were also performed at the 1-year control, because the secondary results were collected at that timepoint, and MRI scans are the most reliable clinical evidence on the severity of OA. Another limitation is that the concentration of PL was not given, and the platelet counts were not provided. This was not done for other trials included in this review either as it was rather complicated—every patient has a unique growth factor and platelet number/ratio after all. It would be easier to perform in PL compared to PRP; perhaps as PL research further expands, a more concentrated effort will be taken to take advantage of that. Interestingly, the effects of the PL injections seemed to last for a full year. This is unlike the results of the studies of Hosseini et al. and Tyrnenopoulou et al., who found 6 months to be the peak of efficacy. Future studies will, therefore, have to ascertain the exact timeframe that would be the most effective in administering PL injections.

The closest analogue to PL is PRP, as it directly derives from it. If PL is found to be more effective than PRP, it could substitute it completely in future. PRP compares similarly to the results shown for PL; at 6 months, it shows a significant difference from baseline for WOMAC, VAS, and KSS scores [33, 34]. In a similar manner to PL, the efficacy seems to drop after 12 months when compared to 6; this drop ranges from moderate to a complete return to baseline [34, 35], suggesting a peak effectiveness at 6 months after the first injection. The effectiveness of PRP injections could depend on the total platelet count. Bansal et al. found that a concentration of 10 billion platelet was necessary for any chondroprotective effects to be visible at the 1-year mark [12]. Research has shown that PRP trials are prone to the placebo effect as mild OA cases tend to see significant improvement in pain scores that are not as evident in moderate-to-severe cases of OA [36]. On the other hand, this may simply suggest that PRP therapy is more effective at earlier stages of OA and that any regenerative abilities decrease with disease progression. The effectiveness of PRP to promote damaged tissue regeneration is still controversial [37]; perhaps, PL treatments will be pushing the efficacy to a point of higher confidence.

When compared to stem cell treatments, another experimental treatment for treating KOA, stem cell injections currently use mesenchymal stem cells derived from different sources (bone marrow, adipose tissue, placental/stromal cells, and synovial tissue) [38]. Stem cell treatments incur a remarkable cost to the patients, with an average price of around $5000 [39]. As an experimental treatment, such treatment is typically not covered by national healthcare services, leaving to the finances most of times on the patient. Benefits of stem cell treatments are contested, and placebo effect may play a large role in treated patietns [39]. The cost itself may create a bias to stem cell providers, as they may seek to find efficacy to promote the treatment. Similarly to PRP and PL injections, randomised controlled trials are still limited [40]. A meta-analysis found that from baseline to 12 months, stem cell injections significantly improved VAS score. However, WOMAC scores for function, pain, and stiffness did not show a significant decrease [41]. At the very least, the results do not currently suggest a noteworthy benefit of stem cell injections over PL treatments. Therefore, based on the cost alone, it would be a boon if PL was found to be more efficacious it as it could lower treatment costs for patients and eventually healthcare systems, if it becomes established as a more conventional therapy.

HA in injections is currently considered the baseline conventional treatment to manage OA [42]. Also known as viscosupplementation, it had its largest meta-analysis performed in 2022. The results showed that overall, the treatment gave marginal benefits over placebo and that it is uncertain if it is justified as a broad treatment [42]. A systematic review of the same year stated that intra-articular HA injections have a “limited role” for the treatment of KOA, but that these are useful for patients that do not find pain relief in analgesic medication and physical therapy, and that are not ready for surgery [43]. Researchers highlighted that HA is more effective when in combination with other treatments, such as PRP, stem cell treatments, and other molecules though further research is necessary. PRP in recent meta-analyses seems to perform better than HA, although both treatments have significant improvements in the 3 and 6 months when compared to control/baseline [44, 45].

Corticosteroid injections are known to perform worse than PRP and HA therapy when it comes to KOA symptom relief [46]. The 2022 NICE guidelines support this finding and state that steroid injections should only be used when other pharmacological treatments are ineffective and that patients should be warned about their short-term relief (< 2 months) [47]. They appear to be well-tolerated when applicable patients are checked well for any contraindications and their potential to derive benefits from the treatment [48]. Overall, evidence is still contested between HA, stem cell, and PRP injections, with no treatment being outright better than the others. Some evidence seems to indicate that PRP may be slightly more effective [49]. PL may have the potential to improve the overall efficacy of injectable treatments for pain relief and regenerative effects and lead the shift in research from analgesic-focused to regenerative-focused.

There are some limitations of the current results. Firstly, there is the possibility that some manuscripts were not included. That is because platelet lysate is still loosely defined with some researchers/physicians calling it “activated PRP” or “lysed PRP”. Although we tried to mitigate this risk by (a) performing the database search for words for the entire article (instead of title + abstract only) and (b) using broad search terms synonyms and wildcards (“*” symbol), there is still the chance that some paper was excluded. Furthermore, given that papers wildly varied in how they collected results, it was not possible to perform any further statistical analysis to analyse the data. Additionally, most studies included subjective measurements for the results, typically through questionnaires assessing the pain of the participants. That means that even if the studies were performed well, limiting bias, there will always be a higher risk of bias just due to the subjectivity in the measurement of such results. Finally due to the studies having a high level of heterogeneity in their reporting of results, no secondary analysis of the compiled results was performed, which limits the inferences that may be drawn from the articles.

## Conclusions

PL is a promising orthobiologic candidate for promoting a regenerative environment in KOA. From preliminary animal and human studies, it would appear to be a viable option in the treatment of knee OA, with studies beginning to prefer it to PRP. Currently, the sparse literature presents encouraging results that should prompt expansion of the topic, with high-level studies to confirm its therapeutic potential.

## Data Availability

The title and abstracts of the reports collected for this review, including their exclusion criteria labelling, are available in Material and Methods.
